# Mechanisms of the Antineoplastic Effects of New Fluoroquinolones in 2D and 3D Human Breast and Bladder Cancer Cell Lines

**DOI:** 10.3390/cancers16122227

**Published:** 2024-06-14

**Authors:** Nicole Ferrario, Emanuela Marras, Veronica Vivona, Federica Randisi, Antonino Nicolò Fallica, Agostino Marrazzo, Gianpaolo Perletti, Marzia Bruna Gariboldi

**Affiliations:** 1 Department of Biotechnology and Life Sciences (DBSV), University of Insubria, 21100 Varese, Italy; nicole.ferrario999@gmail.com (N.F.); emanuela.marras@uninsubria.it (E.M.); veronicavivona98@gmail.com (V.V.); frandisi1@studenti.uninsubria.it (F.R.); gianpaolo.perletti@uninsubria.it (G.P.); 2 Department of Drug and Health Sciences, University of Catania, 95125 Catania, Italy; antonio.fallica93@gmail.com (A.N.F.); marrazzo@unict.it (A.M.)

**Keywords:** anticancer fluoroquinolone derivatives, cell cycle, cell death, anti-migratory effects, topoisomerase II inhibition, HDAC inhibition, triple-negative breast cancer, bladder cancer

## Abstract

**Simple Summary:**

Besides their antibacterial effects, fluoroquinolones possess other valuable properties such as immunomodulatory and anticancer activities. These features make fluoroquinolones ideal compounds for ‘drug repositioning’. In the last decades, fluoroquinolone derivatives with improved anticancer effects have been synthesized. We have previously shown that certain ciprofloxacin and norfloxacin derivatives were more potent anticancer agents than their precursors. For this study, the mechanisms of action of the most active among these derivatives were evaluated in a panel of tumor cell lines, including particularly untreatable cancer forms (e.g., triple-negative breast cancer). The tested compounds exerted several well-known fluoroquinolone effects, like cell cycle arrest, apoptosis induction, migration, and topoisomerase II inhibition, along with rather unexplored mechanisms for fluoroquinolones, namely, alternative cell death and histone deacetylase inhibition. Our results delve deeper into the anticancer mechanisms of fluoroquinolone derivatives that, given their known tolerability, could represent a valid therapeutic option, even in poorly treatable tumors.

**Abstract:**

Antibacterial fluoroquinolones have emerged as potential anticancer drugs, thus prompting the synthesis of novel molecules with improved cytotoxic characteristics. Ciprofloxacin and norfloxacin derivatives, previously synthesized by our group, showed higher anticancer potency than their progenitors. However, no information about their mechanisms of action was reported. In this study, we selected the most active among these promising molecules and evaluated, on a panel of breast (including those triple-negative) and bladder cancer cell lines, their ability to induce cell cycle alterations and apoptotic and necrotic cell death through cytofluorimetric studies. Furthermore, inhibitory effects on cellular migration, metalloproteinase, and/or acetylated histone protein levels were also evaluated by the scratch/wound healing assay and Western blot analyses, respectively. Finally, the DNA relaxation assay was performed to confirm topoisomerase inhibition. Our results indicate that the highest potency previously observed for the derivatives could be related to their ability to induce G2/M cell cycle arrest and apoptotic and/or necrotic cell death. Moreover, they inhibited cellular migration, probably by reducing metalloproteinase levels and histone deacetylases. Finally, topoisomerase inhibition, previously observed in silico, was confirmed. In conclusion, structural modifications of progenitor fluoroquinolones resulted in potent anticancer derivatives possessing multiple mechanisms of action, potentially exploitable for the treatment of aggressive/resistant cancers.

## 1. Introduction

Fluoroquinolones (FQs) are among the largest groups of antibacterial agents used worldwide, with favorable physicochemical and pharmacokinetic properties, such as the excellent bioavailability (levofloxacin: 99%) after oral administration and long half-life (moxifloxacin: 13 h) shown by late-generation compounds [1,2,3]. From a chemical point of view, FQs are characterized as possessing two ionizable centers, specifically a carboxylic acid functional group and a basic nitrogenated heterocycle that confer them a zwitterionic behavior with a neutral net charge at their isoelectric point, usually corresponding to the physiological pH. Therefore, FQs’ solubility and bioavailability are dependent on the chemical environment in which they are found [4], i.e., they are more soluble in polar solvents when ionized; contrarily, they are more lipophilic in their neutral or zwitterionic state, allowing for them to penetrate tissues such as the prostate, kidney, and liver very well. For this reason, they are typically used to treat urinary and genital infections [3,5]. However, modification of their chemical structure allowed for the development of FQs active against Gram-positive infections of the respiratory tract. Inhibition of the catalytic cycle of the enzymes DNA gyrase and topoisomerase IV, which are responsible for the replication and transcription of bacterial DNA, is the main mechanism responsible for the antibacterial action of fluoroquinolones [3,6,7,8]. Recently, a similar effect of FQs was shown on the eukaryotic analog topoisomerase II. This has attracted attention to their potential cytotoxic properties, which can be exploited in anticancer treatment [9,10,11], as shown by several studies performed on colon, breast, lung, and bladder cell lines [12,13,14]. An analogous mechanism of action indeed characterizes several clinically used antitumor agents, such as etoposide, doxorubicin, amsacrine, or mitoxantrone [15,16,17]. Furthermore, several desirable features make fluoroquinolones and their derivatives interesting antineoplastic drugs, namely, reduced toxicity, lower incidence of chemoresistance, decreased rates of drug-induced secondary tumors, and higher potency than other topoisomerase II inhibitors [1,10]. Quinolone derivatives are also characterized by high flexibility and ease of synthesis using different procedures and building blocks, encouraging researchers to prepare and test several versatile chemical structures [11,18,19]. Finally, among other drugs, fluoroquinolones have also proven to be ideal candidates for drug repositioning, viz., the finding of new applications of a known drug beyond its original ones. Therefore, it is believed that repositioning might overcome the limits related to drug development, such as productivity problems, increased costs, regulatory hurdles, and generic drug challenges [20,21]. As compared to traditional drug discovery, drug repositioning allows for a faster and cheaper introduction of drugs into the market and clinical use. A possible obstacle to repurposing may be the toxicity profile of candidate drugs [14,22]. However, although some concerns about fluoroquinolone safety have been reported, which have (i) compelled regulatory agencies to issue specific warnings or (ii) led to the withdrawal of certain molecules from the market [23], the FQs used in the clinics today are known to be generally safe in humans [3,24].

Structural activity relationship (SAR) studies of fluoroquinolones led to the synthesis of clinically relevant analogs optimized for their antibacterial effects [3,25]. Despite several attempts to improve their anticancer effects, the structural requirements related to the optimization of this activity have not yet fully emerged. In general, there seems to be consensus that reducing the zwitterionic character or increasing lipophilicity can result in a better antitumor effect [26,27]. Several derivatives of ciprofloxacin (CIP) and norfloxacin (NOR) have recently been shown to exhibit more potent antitumor activity than the parent compounds in various cancer cell lines [3,14,28]. In this context, our group synthesized a panel of CIP and NOR derivatives, which showed stronger antineoplastic effects in tumor cell lines of different tissue origin, also evidencing a higher extent of interaction with topoisomerase II, compared to the two precursors [29]. Interestingly, the derivatives were also effective in a triple-negative breast cancer (TNBC) cell line, representative of a type of breast cancer particularly resistant to classic treatments. However, no information about the mechanisms involved in the antitumor effects of these derivatives was reported.

Data indicate that fluoroquinolones’ direct action, through topoisomerase II inhibition, leads to cell death, typically by apoptosis, and cell cycle arrest, mainly in the S and G2/M phases [11,30]. Additionally, fluoroquinolones can also hamper cancer growth by indirect effects via modulation of the immune response [1,31]. Furthermore, dysfunction of mitochondria, likely due to the similarity between mitochondrial and bacterial cell structures, has been invoked as a mechanism of action [1,32].

Effects of fluoroquinolones on migration and metastasis have also been reported. In particular, certain FQs can suppress matrix metalloproteinase 9 (MMP9) production [28,33].

In the present work, we confirmed the antiproliferative effects of the CIP and NOR derivatives that have shown higher potency in our previous work, demonstrating them worthy for further studies, on two breast cancer cell lines, MCF7 and MDA-MB231. Furthermore, the effects of these derivatives were also evaluated in another TNBC cell line, namely, MDA-MB453 cells, and in two bladder cancer cell lines (RT112 and 5637), all selected because of the poor therapeutic options available for this type of tumor. Furthermore, other authors have recently reported the interesting activity of FQs in bladder cancer cell lines [1,5].

Recognized anticancer mechanisms of fluoroquinolones, such as the induction of cell cycle arrest and apoptotic cell death, anti-migratory effects, and inhibition of topoisomerase II activity, were investigated. Moreover, new potential mechanisms related to the anticancer activity of the studied fluoroquinolones were evaluated, including their ability to induce necrosis or autophagy and to operate as HDAC inhibitors.

## 2. Materials and Methods

### 2.1. Drugs and Chemicals

**Figure 1:**
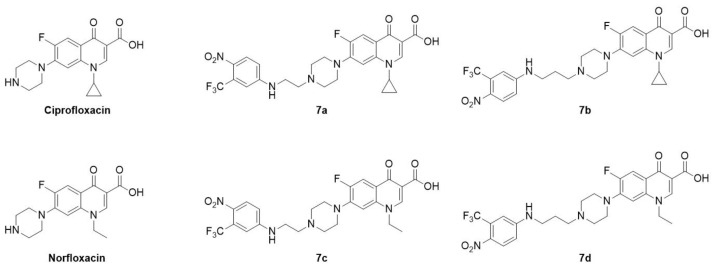
Chemical structure of ciprofloxacin, norfloxacin, and their derivatives, all used in this study.

Ciprofloxacin (CIP), norfloxacin (NOR), and all chemicals and reagents were purchased from Sigma-Aldrich, Merk or Euroclone (Milan, Italy). The synthetic procedures of CIP and NOR derivatives, namely, **7a**,**b** and **7c**,**d**, respectively, were previously reported [29]. These compounds belong to a series of nitric oxide (NO) photodonor hybrids, endowed with a 4-nitro-3-trifluoromethyl-aniline moiety for the light-triggered release of NO [34] and linked to the FQ scaffold through an alkyl bridge of two or three methylene units. The photodonor is able to release NO upon stimulation exerted by a suitable light source. This moiety was selected considering that NO is a gasotransmitter that has a positive role in cancer cell death depending on its concentration in the tumor niche [35] and whose release can be fine-tuned upon light stimulation [36]. Despite the expectations, previous results demonstrated that NO photorelease does not play any role in the antiproliferative effects exerted by these compounds. Chemical structures are summarized in Figure 1.

### 2.2. In Silico Determination of LogP Values

The logarithm of the octanol–water partition coefficient (LogP) for compounds **7a**–**d** was determined in silico using four different software programs. LogP values thus obtained are referred as LogP from Chemicalize (https://chemicalize.com/), miLogP from Molinspiration (https://www.molinspiration.com/), consensus LogP (mean value of iLOGP, XLOGP3, WLOGP, MLOGP, and SILICOS-IT) from SwissADME (http://swissadme.ch/), and average logP (mean of ALogP, and XLOGP2) from the Virtual Computational Chemistry Laboratory (VCCLAB, http://www.vcclab.org/). All websites were accessed on 5–6 June 2024.

### 2.3. Cell Lines and Culture Conditions

MCF7 (human luminal-A adenocarcinoma), MDA-MB231 (human TNBC), MDA-MB453 (human TNBC), RT112 (human grade II urinary bladder transitional carcinoma), and 5637 (HTB-9; human epithelial grade II urinary bladder carcinoma) were originally obtained from the ATCC (American Type Culture Collection, Manassas, VA, USA). WH1 human fibroblasts were kindly provided by Dr. Guven [37]. All the cells were maintained under standard culture conditions (37 °C; 5% CO_2_)—in RMPI1640 medium (MCF7, MDA-MB231, and RT112 cells), DMEM (MDA-MB453 and 5637 cells), or Iscove’s medium (WH1)—supplemented with 10% fetal calf serum, 1% glutamine, and 1% antibiotics mixture; an extra 1% sodium pyruvate and 1% non-essential amino acids were added in DMEM.

MCF7 spheroids were obtained by seeding 2.5 × 10^3^ cells/well into 96 U plates Nunclon Sphera. Spheroids were used on day 7 of growth. RT112 spheroids were instead obtained through the hanging drop technique [38]. Briefly, 2.5 × 10^4^ cells/mL were seeded in 27 µL droplets of medium on 48-well plate lids. To avoid dehydration, each well was filled with PBS. Cells were then incubated in standard conditions. Due to the combination of surface tension and gravitational forces, the drops maintained their shape and remained hanging on the lid, facilitating the formation of spheroids. After 2 weeks, each spheroid was transferred to 96-well plates, whose wells were previously coated with agar, and incubated for 24 h before use.

### 2.4. Cell Viability Assay

The effect of the treatment with **7a**, **7b**, **7c**, and **7d** on MCF7, MDA-MB231, MDA-MB453, RT112, 5637, and WH1 cell viability was evaluated through the MTT (3-(4,5-dimethylthiazol-2-yl)-2,5-diphenyl-2H-tetrazolium bromide) assay [39]. Results were then compared with those obtained by treating the cells with their precursors, namely, CIP and NOR. Only viable cells with an active metabolism and functional mitochondrial succinate dehydrogenase can metabolize MTT to the final product, formazan salts, which can be quantified through colorimetric analysis reading the maximum absorbance at 570 nm.

Briefly, cells were seeded in 96-well plates (2.5 × 10^4^ cells/mL) and grown under standard conditions for 24 h. Cells were then treated with increasing concentrations of compounds under investigation (CIP and NOR 1–100 µM; **7a**, **7b**, **7c**, and **7d** 0.1–10 µM) and incubated for 72 h at 37 °C. At the end of this period, 50 µL of MTT (2 mg/mL in PBS) was added to each well, and plates were incubated for 3h before the solution was discarded and formazan crystals were solubilized in 100 µL of DMSO.

Formazan absorbance was measured at 570 nm with an iMark^TM^ Microplate Reader (Bio-Rad, Segrate, Milan, Italy). The effect of compounds on cell viability was quantified by calculating the IC_50_ values based on non-linear regression analysis of dose–response curves, performed using CalcuSyn software 1.1.4 (Biosoft, Cambridge, UK).

The effects on MCF7 and RT112 spheroids were assessed based on the evaluation of spheroids’ growth, by their observation through an inverted microscope, and cell viability through the trypan blue exclusion assay [40]. Briefly, spheroids were treated with concentrations of the studied compounds corresponding to the IC_50_ values obtained by the MTT assay on 2D-cultured MCF7 and RT112 cells and incubated at 37 °C for 72 h. Control spheroids were incubated with culture medium for the same amount of time. At the end of this period, 3/5 spheroids for each treatment were collected independently, disaggregated using trypsin–EDTA solution, and live cells were counted using a Burker hemocytometer, following trypan blue staining. Percentages of apoptotic cells were also evaluated (see Section 2.4). Pictures of the same spheroids were taken using a camera connected to an Olympus IX8I microscope (Olympus Italia, Segrate, Milan, Italy) immediately before (0) and at the end of treatment (72 h).

### 2.5. Cell Death Induction

The ability of CIP and NOR derivatives to induce apoptosis, necrosis, and/or autophagy was evaluated by flow cytometry, following staining with propidium iodide (apoptosis and necrosis), and by Western blot analysis of the autophagic marker LC3-II (autophagy), as previously shown [41]. For apoptotic and necrotic cell death evaluation, cells were seeded in 12-well plates (6 × 10^4^/mL) and allowed to grow in a CO_2_ incubator at 37 °C. After 24 h, cells were treated for 72 h with concentrations of CIP, NOR, and their derivatives corresponding to the respective IC_50_ values (Table 1). To evaluate the percentage of apoptotic cells, at the end of treatment, cells were harvested, washed in PBS, and fixed in 70% ethanol at −20 °C. After a further wash in PBS, DNA was stained with a solution of PI in PBS (50 µg/mL) in the presence of RNAse (RiboNucleicAcid hydrolase) (30 U/mL) at room temperature for 15 min. Samples were then analyzed using a FACSCalibur Becton Dickinson flow cytometer (Becton Dickinson, Milan, Italy) equipped with an air-cooled argon ion laser (15 mW, 488 nm), using CellQuestPRO 6.0 software (Becton Dichinson). The fluorescent emission of PI was collected through a 575 nm bandpass filter and the percentage of apoptotic cells in each sample was determined based on the sub-G1 peaks detected in monoparametric histograms acquired in log mode. Evaluation of apoptotic cells from spheroids was performed following the same protocol after spheroid disaggregation.

Evaluation of necrotic cells was performed with the aforementioned procedure by omitting the fixation step. In this way, PI enters only membrane-damaged (i.e., necrotic) cells.

To evaluate LC3-II protein levels, cells were seeded into 100 mm Petri dishes (1.2 × 10^6^ cells/Petri) and allowed to grow for 24 h before treatment with the same concentrations of the studied compounds used to evaluate apoptosis and necrosis. After 72 h, cells were processed as previously described [42]. The obtained total cell proteins were collected, protein content was quantified through the BCA assay kit, and 60 µg of proteins were loaded on a polyacrylamide gel (15%) and separated under denaturing conditions. Proteins were then transferred to a nitrocellulose membrane(Sigma Aldrich, Merk) using a transblot machinery, Bio-Rad (2 h, 400 V, 200 mA), and a wet transfer buffer (25 mM Tris, 192 mM glycine, 15% MeOH). Membranes were first incubated for 45 min in a blocking solution (milk 5% and PBS-TWEEN 0.1%), to block non-specific sites, and then overnight with rabbit polyclonal antibody anti-LC3-II (Sigma Aldrich, Merk). Thereafter, membranes were washed in PBS-TWEEN 0.1% and incubated with a secondary anti-rabbit antibody conjugated to horseradish peroxidase. After further washing steps, immunoreactive bands were visualized through chemiluminescence analysis by using the Bio-Rad ChemiDoc^TM^ Imaging System, with the aid of a detection solution comprised in the kit Westar Supernova (Cyangen, Bologna, Italy). To verify the homogeneity of the samples loaded on the gel and to ensure that the variations observed in the proteins of interest were not due to a generalized alteration of the protein synthesis or incorrect loading of the samples, the membranes were co-incubated with a primary antibody directed against actin (Santa Cruz Biotechnology, DBA, Segrate, Milan, Italy)). Densitometric analyses were performed by using Image Lab 6.1 software (Bio-Rad).

### 2.6. Effects on Histone Acetylation

Possible effects of the studied compounds on the activity of histone deacetylase (HDAC) enzymes were evaluated by analyzing the levels of the acetylated form of histone 3 and 4, through Western blot analysis, as described in Section 2.5 [43]. For this set of experiments, mouse monoclonal anti-acetylated histone H3 and histone H4 antibodies (Santa Cruz Biotechnology) and anti-mouse conjugated to horseradish peroxidase were used as primary and secondary antibodies, respectively.

### 2.7. Effects on Cell Migration

The effects of **7a**, **7b**, **7c**, and **7d** and of their progenitors eventually induced on cell migration were evaluated through the scratch/wound healing assay [41]. Preliminary tests highlighted the inefficient migration of MCF7 cells, excluded from this set of experiments.

Cells were seeded into 12-well plates (MDA-MB231 and 5637: 4 × 10^4^ cells/mL; MDA-MB453 and RT112: 2 × 10^4^ cells/mL) and allowed to grow up to approximately 100% confluence. A scratch was then made with a pipette tip, the wells were washed with fresh PBS to eliminate detached cells, and cells were treated with subtoxic concentrations of the tested compounds, corresponding to their respective IC_10_. Pictures of the scratch wound were taken immediately after the treatment (t0) and when the scratches in control samples (i.e., not treated) were closed (15 or 24 h after) using a camera connected to an Olympus IX81 inverted microscope. Percentages of open scratches were determined using TScratch 1.0 software.

Possible effects of the studied compounds on metalloproteinases 2 and 9 (MMP2 and MMP9) were evaluated by Western blot analysis, performed as previously described but treating the cells with the same subtoxic concentrations used for the Scratch Wound Healing assay. To detect MMP2 and MMP9 protein levels, rabbit polyclonal anti-MMP2 and anti-MMP9 antibodies (Santa Cruz Biotechnology) were used.

### 2.8. Effects on DNA Relaxation

A DNA relaxation assay was performed to indirectly evaluate topoisomerase activity, using nuclear extracts obtained from MCF7 and RT112 cells [44]. Briefly, cells were seeded in Petri dishes (1 × 10^7^) and grown until 80% of confluence. Thereafter, cells were harvested, counted, washed in cold PBS, and resuspended in 20 µL Buffer A (Hepes pH 7.9 10 mM, MgCl_2_ 1.5 mM, KCl 10 mM, DTT 0.5 mM, PMSF 0.5 mM in water)/10^7^ cells. To allow for cell lysis, samples were then incubated in ice and centrifuged, and supernatants, representing the cytoplasmic extract, were discarded. Instead, the pellets were resuspended in 15 µL Buffer B (Hepes pH 7.9 10 mM, glycerol 25%, MgCl_2_ 1.5 mM, NaCl 0.42 M, EDTA 0.2 M, DTT 0.5 mM, PMSF 0.5 mM, H_2_O)/10^7^ cells. Again, cells were centrifuged and the supernatants, representing the nuclear extracts, were transferred in a 1.5 mL tube and diluted in 40 µL Buffer C (Hepes 20 mM, glycerol 20%, KCl 0.05 M, EDTA 0.2 mM, DTT 0.5 mM, PMSF 0.5 mM, H_2_O)/10^7^ cells. Dosage of protein content was performed using a BCA assay, and nuclear extracts were stored at −80 °C.

In the relaxation assay, the substrate is supercoiled pBR322, which is relaxed by topoisomerase II. For the assay, the following reaction mixture was prepared (20 µL total volume): 7 µg of pBR322 purified DNA plasmid (Euroclone, Pero, Milan, Italy), 2 µL assay buffer 10× (50 mM Tris HCl (pH 7.5), 125 mM NaCl, 10 mM MgCl_2_, 5 mM DTT, and 100 μg/mL albumin), 1 mM ATP (Thermo Fisher Scientific, Monza, Milan, Italy), and 4 μg of nuclear extracts, in the presence of 10 µM or 100 µM of CIP, NOR, and their derivatives. Samples containing > 10% DMSO were used as positive controls.

Mixture reactions were incubated at 37 °C for 30 min. Therefore, 4 µL of 6x loading dye (10 mM Tris-HCl (pH 7.6) 0.03% bromophenol blue, 60% glycerol, 60 mM EDTA) was added. A 24 µL quantity of the resulting solution was loaded on 1.5% agarose gel and samples were resolved by electrophoresis for 2 h at 85 V, in TAE buffer (0.4 M Tris-Acetate and 0.01 M EDTA pH 8.3). Electrophoresis gels were incubated for 20 min with ethidium bromide (1 µ/mL) and visualized by UV light.

### 2.9. Statistical Analysis

The null hypothesis for all comparisons was the absence of intergroup differences for each tested variable (equal variances). Intergroup analysis was performed by one-way ANOVA and the Bonferroni correction test for multiple comparisons. The upper threshold for an alpha error probability was set to 5%. All statistical analyses were performed using the GraphPad PRISM 8.4 software.

## 3. Results

### 3.1. CIP and NOR Derivatives Affect Human Breast and Bladder Cancer Cell Viability in 2D and 3D Models

The effects of **7a**, **7b**, **7c**, and **7d** on the viability of MCF7, MDA-MB231, and MDA-MB453 human breast cancer cells and RT112 and 5637 human bladder cancer cells were assessed through the MTT assay following 72 h of treatment with increasing concentrations of the drugs. IC_50_ values, obtained from the corresponding dose–response curves, are reported in Table 1. IC_50_ values of the four fluoroquinolone derivatives were compared to those obtained for the reference compounds CIP and NOR in the same experimental conditions, also reported in Table 1. All derivatives were significantly more potent than their respective progenitor, as lower concentrations of drugs were necessary to induce the same effects on cell viability, as demonstrated by the significantly lower IC_50_ values obtained for **7a**, **7b**, **7c**, and **7d** compared to CIP or NOR. Specifically, as indicated in Supplementary Figure S1, which reports potency indexes of the derivatives, **7a** and **7b** were 1.9–7.5- and 2.9–6.99-fold more potent than CIP, respectively, whereas **7c** and **7d** were 5.6–18- and 3.25–16-fold more potent than their NOR precursor, respectively.

The effects of CIP and NOR derivatives were also evaluated in 3D spheroids obtained from MCF7 and RT112 cells, the only lines that were able to grow as spheroids under the experimental condition used, following 72 h treatment with concentrations of the drugs corresponding to the IC_50_ values obtained in monolayer-cultured cells.

**Figure 2:**
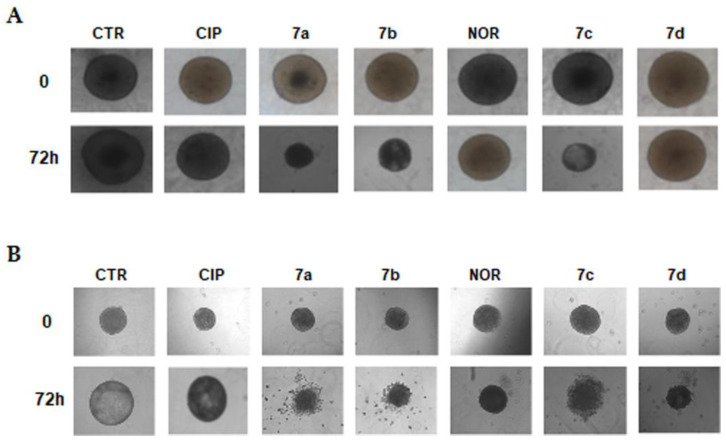
Representative images of MCF7 (**A**) and RT112 (**B**) spheroids following treatment with CIP, NOR, and their derivatives for 72 h at concentrations corresponding to the IC_50_ values obtained in monolayer-cultured cells. Pictures were taken before (0) and at the end of the treatment (72 h) using a camera connected to an Olympus IX8I microscope (magnification 10×).

Images shown in Figure 2 indicate that, morphologically, both MCF7 and RT112 control spheroids increased their size during a 72 h incubation. Treatment with FQ derivatives, with the exception of **7d** in MCF7 spheroids, promoted a substantial decrease in their size after 72 h. Conversely, treatment with CIP and NOR induced only slight changes in spheroid dimensions.

**Figure 3:**
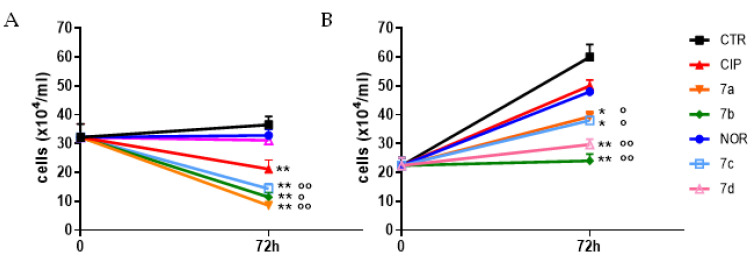
Growth curves of MCF7 (**A**) and RT112 (**B**) spheroids following 72 h treatment with the studied compound and their progenitors at concentrations corresponding to the IC_50_ values obtained in monolayer-cultured cells. Counts of viable cells were performed immediately before treatment (time 0) and 72 h later (mean ± SE of 3/5 spheroids; * *p* < 0.05, ** *p* < 0.01 vs. CTR; ° *p* < 0.05, °° *p* < 0.01 vs respective progenitor).

The observed alterations in spheroids’ growth were also quantitatively confirmed by counting viable cells obtained upon spheroid disaggregation after 72 h treatment. Results reported in Figure 3 indicate that, with the exception of **7d** in MCF7, all derivatives promoted a statistically significant decrease in cell number in spheroids from both cell lines. On the other hand, among the reference compounds, only CIP significantly reduced viable cell numbers in MCF7 spheroids. Therefore, almost all FQ derivatives showed to be significantly more potent in inhibiting spheroid growth, compared to their respective reference compounds.

### 3.2. CIP and NOR Derivatives Induce S-G2/M Cell Cycle Arrest and Apoptotic and Necrotic Cell Death in 2D and 3D Models

The ability of **7a**, **7b**, **7c,** and **7d** derivatives to induce cell cycle alterations and apoptotic and/or necrotic cell death in breast and bladder cancer cells was evaluated through flow cytometric analysis following 72 h treatment with equitoxic concentrations of each compound. Results were compared with those obtained by treating cells with CIP and NOR.

**Figure 4:**
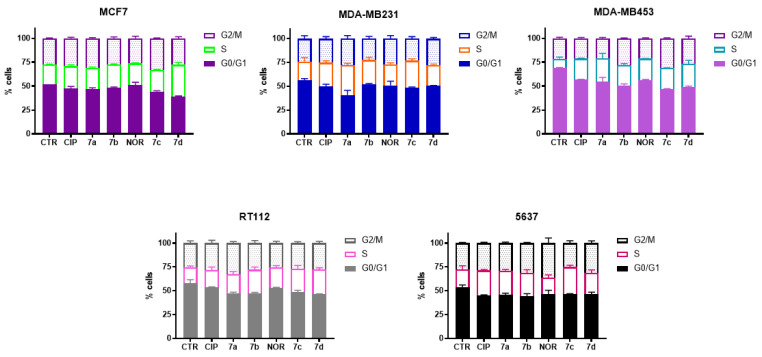
Cell cycle distribution of MCF7, MDA-MB231, MDA-MB453, RT112, and 5637 cells following 72 h treatment with CIP, NOR, and their derivatives at concentrations corresponding to the IC_50_ values reported in Table 1 (mean ± S.D. of three independent experiments).

Figure 4 shows that treatment with the four derivatives, as well as with their precursors, induced cell cycle arrest at S/G2M phase in all cell lines.

**Figure 5:**
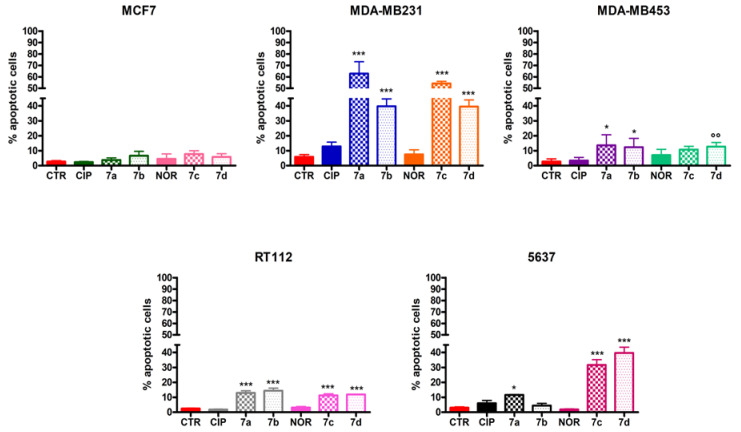
Percentage of apoptotic MCF7, MDA-MB231, MDA-MB453, RT112, and 5637 cells following 72 h treatment with CIP, NOR, and their derivatives at concentrations corresponding to the IC_50_ values reported in Table 1 (mean ± S.D. of three independent experiments; * *p* < 0.05, *** *p* < 0.001 vs. CTR and CIP or NOR; °° *p* < 0.01 vs. CTRL).

Differently from CIP and NOR, which did not promote apoptosis in any of the investigated cell lines, the derivatives induced apoptotic cell death to different extents in MDA-MB231, MDA-MB453, RT112, and 5637 cell lines. Specifically, all derivatives significantly induced apoptosis in MDA-MB231 and RT112 cells, with higher percentages of apoptotic cells observed in the former. At the same time, only **7a**, **7b**, and **7d** were able to trigger apoptotic cell death in MDA-MB453, while **7a**, **7c**, and **7d** were the most active compounds in 5637 cells. None of the derivatives induced apoptosis in MCF7 cells (Figure 5).

**Figure 6:**
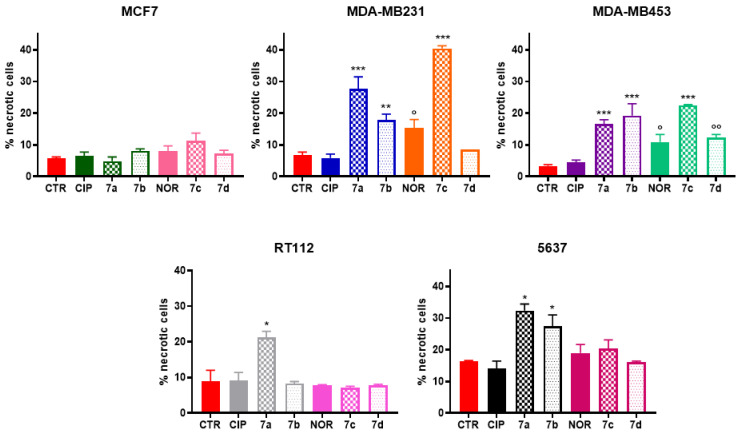
Percentage of necrotic MCF7, MDA-MB231, MDA-MB453, RT112, and 5637 cells following 72 h treatment with CIP, NOR, and their derivatives at concentrations corresponding to the IC_50_ values reported in Table 1 (mean ± S.D. of three independent experiments; * *p* < 0.05, ** *p* < 0.01; *** *p* < 0.001 vs. CTR and CIP or NOR; ° *p* < 0.05, °° *p* < 0.01 vs. CTRL).

Similarly, Figure 6 shows that only **7a**, **7b**, **7c**, and **7d** triggered necrosis to different extents in MDA-MB231, MDA-MB453, RT112, and 5637 cells.

In particular, all derivatives could promote necrotic cell death in MDA-MB453 cells. The same applied to MDA-MB231, with the exception of compound **7d,** which was not able to induce necrosis. Moreover, **7a** and **7b** derivatives fostered necrosis in 5637 cells, while only **7a** induced the same effect in RT112 cells. On the other hand, among the precursor molecules, only NOR induced an increase in the percentage of necrotic cells exclusively in the two TNBC cell lines. Again, MCF7 cells did not respond to the treatment by undergoing necrotic cell death.

**Figure 7:**
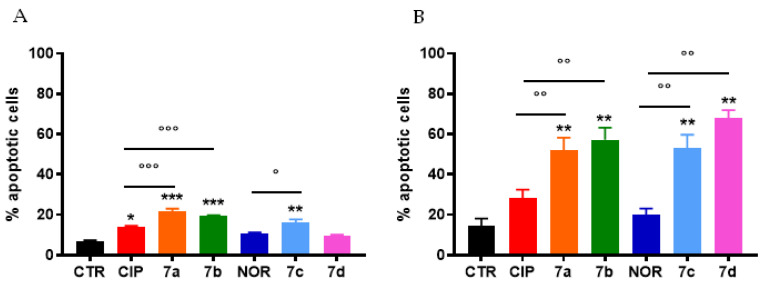
Percentage of apoptotic cells from MCF7 (**A**) and RT112 (**B**) spheroids following 72 h treatment with CIP, NOR, and their derivatives at concentrations corresponding to the IC_50_ values reported in Table 1 (mean ± S.D. of three independent experiments; * *p* < 0.05, ** *p* < 0.01, *** *p* < 0.001 vs. CTR; ° *p* < 0.05, °° *p* < 0.01, °°° *p* < 0.001).

Percentages of apoptotic cells were also evaluated in cells obtained from MCF7 and RT112 spheroids. Generally, higher levels of apoptosis were observed in cells derived from spheroids compared to MCF7 and RT112 cells grown in monolayer. However, qualitatively, similar results to those obtained in monolayer-maintained cells were observed (Figure 7). Specifically, also in spheroids CIP and NOR derivatives were more effective in inducing apoptotic cell death than their respective progenitors. In particular, only FQ derivatives induced a significant increase in the percentages of apoptotic cells in RT112 spheroids, while **7a**, **7b**, as well as their progenitor CIP and **7c** could induce apoptosis in MCF7 spheroids.

### 3.3. CIP and NOR Derivatives Induce Autophagy in MCF7 Cells

**Figure 8:**
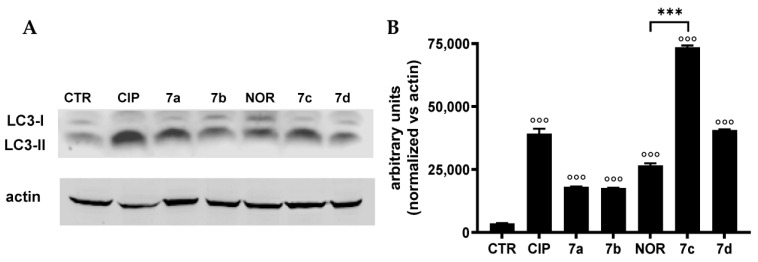
LC3-II protein levels ((**A**) representative Western blot analysis out of two independent experiments with similar results) in MCF7 cells treated 72 h with CIP, NOR, and their derivatives at concentrations corresponding to the IC_50_ values reported in Table 1, and relative densitometric analysis performed in all Western blot experiments (**B**) (mean ± S.D. of two independent experiments; °°° *p* < 0.001 vs. CTR; *** *p* < 0.001). The uncropped bolts are shown in Supplementary Materials.

The possible induction of autophagy in MCF7 cells, which did not undergo apoptotic or necrotic cell death following treatment with CIP, NOR, and their derivatives, was assessed by Western blot analysis. The levels of the autophagosomal marker LC3-II, whose overexpression is strictly correlated to the activation of the autophagic process, were measured. Results are shown in Figure 8 and indicate that all tested compounds were able to increase LC3-II levels in MCF7 cells, likely indicating the induction of autophagy.

### 3.4. CIP and NOR Derivatives Inhibit Cell Migration by Reducing MMP2 and MMP9 Protein Levels

Migration is a key step in the metastatic process. For this reason, it is pivotal to investigate cancer cell motility after treatment to understand whether any compound can act as anti-migratory agent.

To evaluate the effect of the studied compounds on cell migration, the scratch/wound healing assay was performed on cell lines that had shown intrinsic migratory capability in preliminary studies, namely, MDA-MB231, MDA-MB453, RT112, and 5637 cells. MCF7 cells do not possess this characteristic and were excluded from this set of experiments. To exclude that the possible inhibition of cancer cell migration was a consequence of the cytotoxic effect of CIP, NOR, and their derivatives, we defined the appropriate concentrations of these compounds that showed only a subtoxic effect on cell viability.

**Figure 9:**
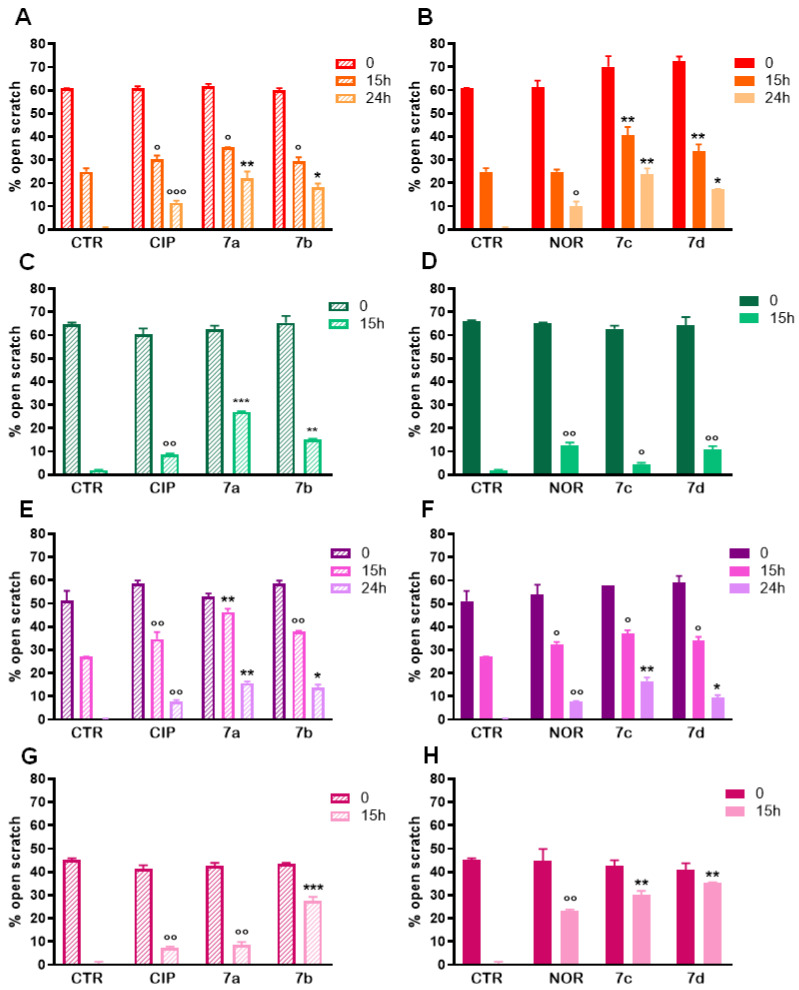
Percentages of open scratches in MDA-MB231 (**A**,**B**), MDA-MB453 (**C**,**D**), RT112 (**E**,**F**), and 5637 (**G**,**H**) cells following treatment with **7a**, **7b**, **7c**, and **7d** and their precursors CIP or NOR (mean ± S.D. of three independent experiments; * *p* < 0.05; ** *p* < 0.001; *** *p* < 0.001 vs. CTR and CIP or NOR; ° *p* < 0.05; °° *p* < 0.01; °°° *p* < 0.001 vs. CTR).

Pictures of the scratches taken at 15 h and 24 h of treatment (Figure S1) show that FQ derivatives, as well as CIP and NOR, reduced cell proliferation and migration in all cell lines under investigation. To better characterize these observations, quantification of the percentages of open scratches was also performed using T-Scratch software 1.0, and results confirmed what was visually assessed in the pictures: except for MDA-MB231 cells exposed for 15 h to NOR, all compounds significantly reduced cellular migration in all cell lines when compared to controls, as indicated by the statistically significant increase in the percentage of open scratches (Figure 9).

**Figure 10:**
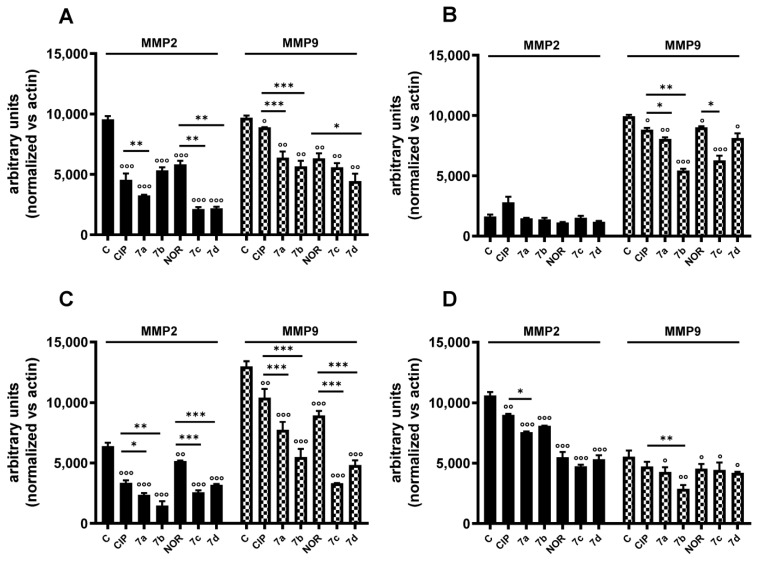
MMP2 and MMP9 protein levels in MDA-MB231 (**A**), MDA-MB453 (**B**), RT112 (**C**), and 5637 (**D**) cells following treatment with **7a**, **7b**, **7c**, and **7d** and their precursors CIP or NOR. Densitometric analysis was performed using ImageJ 1.53e software (mean ± S.D. of two independent experiments; ° *p* < 0.05; °° *p* < 0.01; °°° *p* < 0.001 vs. CTR; * *p* < 0.05, ** *p* < 0.01, *** *p* < 0.001). Representative Western blots are reported in Figure S2.

Western blot analyses were then carried out to investigate the effects of **7a**, **7b**, **7c**, **7d** and their precursors on the expression of MMP2 and MMP9, which are essential proteins involved in migratory and metastatic processes and which could be involved in the anti-migratory activity observed. Figure S2 and Figure 10 show representative Western blot analysis images and densitometric analysis, respectively, of all experiments related to the aforementioned protein levels after 72 h treatment with the same concentrations of the tested compounds used for the scratch/wound healing assay. In densitometric analysis, MMP2 and MMP9 values were normalized vs. actin. In general, all compounds reduced MMP2 or/and MMP9 levels; however, they reduced MMP2 levels to a greater extent compared to MMP9 ones. Specifically, MMP2 densitometric analysis highlighted that both derivatives and progenitors induced a significant reduction in this protein levels in MDA-MB231, RT112, and 5637 cells, while no alterations were observed in MDA-MB453 cells. Differently from what was observed for MMP2, the FQ derivatives, along with their precursors, significantly decreased MMP9 protein levels in MDA-MB231, MDA-MB453, and RT112 cells. Differently, in 5637 cells they did not show any effect on MMP9 levels. Interestingly, in most cases, derivatives were significantly better metalloproteinase inhibitors than their precursors.

### 3.5. CIP and NOR Derivatives Inhibit HDAC Activity

**Figure 11:**
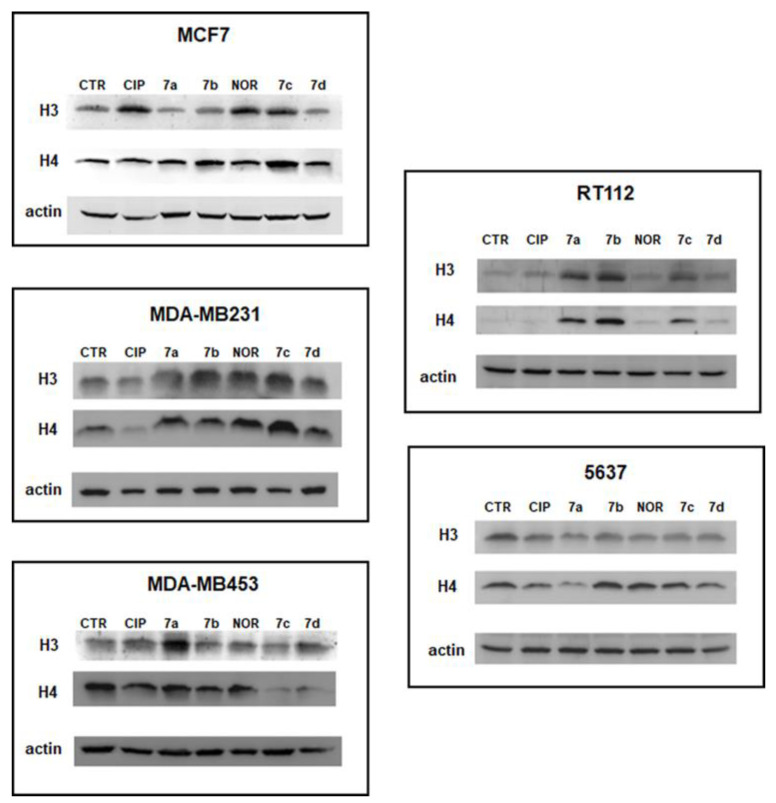
Representative Western blot analyses of acetylated histones H3 and H4 following 72 h of treatment with the studied compounds at concentrations corresponding to the IC_50_ values reported in Table 1. Relative densitometric analysis performed in all Western blot experiments are reported in Figure S3.

Several recent anticancer agents have been shown to act as histone deacetylase (HDAC) inhibitors, thus exerting their effect by influencing epigenetic and non-epigenetic pathways inducing cell death and cell cycle arrest in cancer cells [45]. To investigate the capability of equitoxic concentrations (corresponding to IC_50_ values) of CIP, NOR, and their derivatives to act as HDAC inhibitors in the studied cell lines, Western blot analyses of the acetylated form of histone H3 and histone H4 were performed after 72 h of treatment. Results are reported in Figure 11 and Figure S3, which show representative Western blot results and densitometric and statistical analyses of all the experiments performed, respectively.

The data reported here indicate that all derivatives promoted a statistically significant increase in acetylated histone H3 and/or H4 in most cell lines. Specifically, in MCF7 and RT112 cells, all derivatives induced an increase in acetylated H4 protein levels. In contrast, the increase in H3 levels in these two cell lines was observed only following treatment with **7c** and **7d** in the former cell line and **7a**, **7b**, and **7c** in the latter. In MDA-MB231 cells, both forms of acetylated histones, and H3 levels were induced by compounds **7a**–**c**. Only **7a** and **7d** increased acetylated H3 in the MDA-MB453 cell line, and **7b** acetylated H4 in 5637 cells.

On the other hand, CIP and NOR only increased acetylated H4 levels in MCF7 and in MDA-MB231 and 5637 cells, respectively. Interestingly, derivatives were better than their progenitors in most of the cases.

For a simpler observation of these results, Table S2 summarizes the ability of **7a**–**d**, CIP, and NOR to increase H3 and/or H4 protein levels in the five cell lines considered.

### 3.6. CIP and NOR Derivatives Inhibit Topoisomerase-Dependent DNA Relaxation

**Figure 12:**
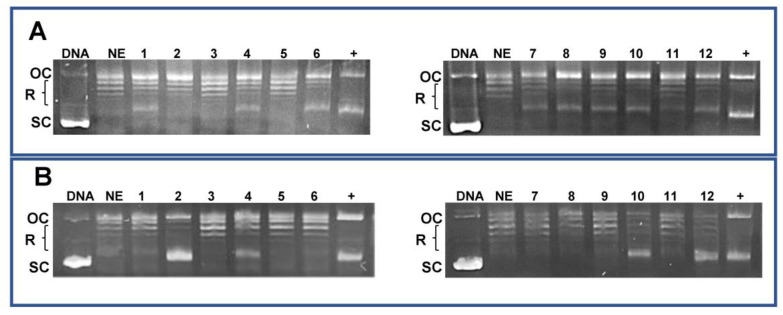
DNA relaxation assay for topoisomerase activity in MCF7 (**A**) and RT112 (**B**) nuclear extracts (OC: nicked, open circular DNA; R: relaxed DNA; SC: supercoiled DNA. Lines legend: DNA: pBR322 supercoiled plasmid; NE: nuclear extracts; **1**: NE + CIP 10 µM; **2**: NE + CIP 100 µM; **3**: NE + **7a** 10 µM; **4**: NE + **7a** 100 µM; **5**: NE + **7b** 10 µM; **6**: NE + **7b** 100 µM; **7**: NE + NOR 10 µM; **8**: NE + NOR 100 µM; **9**: NE + **7c** 10 µM; **10**: NE + **7c** 100 µM; **11**: NE + **7d** 10 µM; **12**: NE + **7d** 100 µM; +: positive control (DMSO > 10%). The uncropped bolts are shown in Supplementary Materials.

In our previous work, molecular modeling studies investigating the interaction with bacterial and cellular targets showed that, compared to CIP and NOR, all derivatives have higher in silico affinity—with lower calculated free energies of binding—toward topoisomerase II-alpha (TopoIIα) [29]. Here, the effects of FQ derivatives and their progenitors on human topoisomerase activity, in breast and bladder cancer cells, were investigated through the DNA relaxation assay, which relies on using a supercoiled plasmid that mimics genomic constraints of DNA [46]. Nuclear extracts obtained from MCF7 and RT112 cell lines were used. Results summarized in Figure 12 confirm that derivatives are in general better topoisomerase inhibitors than CIP and NOR, as shown by the increased presence of supercoiled and/or nicked, open circular DNA. However, FQ derivatives acted to different extents in breast and bladder nuclear extracts. Specifically, when nuclear extracts obtained from MCF7 cells were used, **7a**–**b** and **7c**–**d** inhibited DNA relaxation at both drug concentrations tested (namely, 10 and 100 µM), while only the highest concentration of **7a** and **7c**–**d** exerted the same effects in RT112 nuclear extracts. Only a slight reduction in topoisomerase action was observed following incubation with CIP in extracts of both cell lines and with NOR in MCF7 extracts.

## 4. Discussion

Fluoroquinolones are the largest class of antimicrobial agents used worldwide [3,47]. Apart from their antibacterial efficacy, several other effects have also been reported for FQs, such as antiprotozoan, immunomodulatory, and anticancer properties, some of which are under preclinical investigation, and some that are being evaluated in the frame of clinical trials [3,20].

Based on SAR studies, structural modifications of present-day fluoroquinolone molecules have been performed to redeploy them from antibacterial agents to highly effective anticancer molecules [1,3,20]. Results of previous work from our group showed enhanced anticancer potency of novel CIP and NOR derivatives, compared to their progenitor compounds, which was explained considering the specific chemical modifications in the original structure that have probably enhanced the derivatives’ cytotoxic action against cancer cells. However, no information about the possible mechanisms of action of the derivatives has been provided so far [29]. In the present work, the most potent CIP and NOR derivatives synthesized by our group, namely, **7a**, **7b**, **7c**, and **7d**, were selected and tested on a panel of breast (including two TNBC) and bladder cancer cell lines. Potential mechanisms of action of the FQ derivatives were also investigated and results were compared to those obtained with their precursor molecules.

The higher potency of the novel derivatives, compared to CIP and NOR, was confirmed in two of the three breast cancer cell lines used, which had been already tested in our previous work (MCF7 and MDA-MB231 cells). Furthermore, the FQ derivatives also demonstrated their higher potency in an additional TNBC cell line, MDA-MB453, and in the RT112 and 5637 bladder cancer cell lines. Consistently with our previous data, compound **7c** was the best performing among all derivatives [29].

Minor to moderate toxic effects were also observed in the fibroblast cell line WH1 [29]; however, all derivatives showed higher potency on the cancer cell lines tested, compared to the fibroblast one. Interestingly, the derivatives’ better performance was also confirmed in RT112 and MCF7 spheroids, in agreement with the evidence reported by other authors, who documented the antiproliferative effects of fluoroquinolones on this 3D model [5,48,49]. These results are exciting, considering that spheroids represent an in vitro model that resembles in vivo tumor architecture and biology, mimicking the main features of solid neoplasia such as hypoxia, nutrient gradient, and cell–cell as well as cell–microenvironment interactions. Thus, spheroids represent an improved model compared to 2D monolayer cell cultures [50,51].

For all new derivatives, we performed an in silico determination of their LogP values using different free software available online. Results (Table S3) showed that compounds **7a**–**d** are more lipophilic compared to their progenitors CIP and NOR, as the structural modification at the nitrogen atom of the C7 heterocycle reduced the zwitterion character of the molecules. This finding seems to confirm the previously reported assumption that linked a strong antitumoral effect when FQs lipophilicity is augmented, whereas an opposite trend was observed for hydrophilic FQs with antibacterial effects and lack of cytotoxicity [52,53].

The anticancer activity of fluoroquinolones may potentially occur through the inhibition of mitochondrial DNA synthesis, resulting in mitochondrial injury, respiratory chain disorders, and depletion of intracellular ATP stores. This may induce cell cycle arrest in S and/or G2/M phases, thus possibly favoring apoptosis [54]. In agreement with the last statement, we observed an S-G2/M-phase cell cycle arrest following treatment with all derivatives, as well as CIP and NOR.

As previously mentioned, fluoroquinolone antibacterials have been shown to induce apoptotic cell death in a variety of tumor cell lines such as breast, colorectal, cervical, bladder, and prostate cancer [20,48,54,55,56]. Our results only partially agree with this assertion. As a matter of fact, beyond our experimental conditions, all derivatives induced apoptotic cell death, while CIP and NOR did not trigger apoptosis. Furthermore, MCF7 cells did not undergo apoptosis at all. This behavior can be explained considering that MCF7 cells are known to lack caspase 3 due to a 47 bp deletion within exon 3 of the CASP-3 gene that causes exon skipping and introducing a premature stop codon, thus not allowing for protein translation [57]. When necrotic cell death was studied, we observed that all derivatives induced necrosis better than their respective precursors in TNBC and bladder cancer cells. These results are quite novel, taking into account that only scarce evidence on necrosis induction after quinolone treatment is reported in the literature [1]. Similar to apoptosis, MCF7 cells did not undergo necrosis at all. However, these cells responded to the treatment with all the tested compounds by activating autophagy. These results can be probably explained by considering the intricate interplay existing between autophagy and apoptosis. Indeed, it has been demonstrated that autophagy activation can reduce apoptosis initiation by downregulating the multitude of pro-apoptotic proteins, or vice versa, apoptosis can be triggered when autophagy can no longer sustain severe cell damage [58]. Furthermore, a recent study showed that the third-generation fluoroquinolone enoxacin can indeed induce autophagy [59].

Interestingly, all FQ derivatives promoted apoptosis in RT112 spheroids and, surprisingly, except for **7d**, also in MCF7 cell spheroids. We do not know the reasons why apoptosis was observed in spheroid-grown but not in 2D-cultured MCF7 cells; however, a similar behavior was reported by Guzman and coworkers in other breast cancer cell lines [60]. Unlike their derivatives, CIP and NOR did not induce substantial changes in spheroid growth and morphology. This last result is in contrast with the findings of other authors, who demonstrated the antiproliferative and pro-apoptotic action of CIP on human T24 bladder cancer spheroids, by observing a reduction in spheroid size and the induction of apoptosis [5]. Nevertheless, higher concentrations of fluoroquinolones were used in that work, compared to those used in the present study.

Cellular migration and invasion are the main features of tumor biology and play a pivotal role in metastasis, which is a major determinant of oncological patients’ death [61]. Published studies reported the anti-migratory nature of quinolone compounds in different cell lines, an effect related to their ability to inhibit metalloproteinase expression [28,33,62]. In agreement with these studies, our results highlighted that, similarly to CIP and NOR, their derivatives significantly inhibited cell migration and induced a general decrease in MMP2 and MMP9 protein levels, with better performances against MMP2 (except for MDA-MB453 cells). Thus, the downregulation of these two proteins seems to be one of the potential mechanisms through which the derivative compounds exert their anti-migratory function, rendering them quite interesting antineoplastic agents for the treatment of highly aggressive and metastatic tumors, such as TNBC.

A relatively novel mechanism of action of FQs evidenced in this study was the observation that the FQ derivatives can induce different extents of upregulation in acetylated forms of histones H3 and H4 in the studied cell lines, thus indicating their possible role as HDAC inhibitors (HDACi). It is well known that histone deacetylases (HDACs), responsible for epigenetic modification of histones, are overexpressed in several tumors and that HDAC inhibitors can induce cancer cell death through cell cycle arrest, apoptosis, or autophagy [45,63]. Thus, evaluating the acetylated form of histones H3 and/or H4 is considered a good strategy to assess HDAC activity and check whether a candidate drug can act as an HDACi [64,65]. To date, HDAC inhibitory activity has been demonstrated only in FQ hybrids and FQs/HDACi conjugates [66,67]. Interestingly, CIP and NOR increased only acetylated H4 levels in MCF7 and in MDA-MB231 and 5637 cells, respectively.

Finally, results obtained in the relaxation assay, in which the ability of the studied compounds to inhibit DNA relaxation is related to their activity on topoisomerase enzymes, confirmed that all derivatives inhibit topoisomerase activity. Further studies may completely clarify the effects of these new fluoroquinolones on topoisomerase II and further investigate whether they act as enzyme catalytic inhibitors or poisons; however, also considering the results obtained in silico in our previous work, the pieces of evidence reported indicate that the antiproliferative activity of the derivatives may be, at least in part, attributed to their effects on TopoII.

Taken together, our results indicate that the FQ derivatives tested in this study may exert their cytotoxic effect through a variety of mechanisms, which make them attractive candidates for future in vivo studies in xenograft and/or carcinogen-induced models. In such in vivo studies, the safety profile of these novel compounds ought to be taken into consideration and thoroughly investigated. The CIP and NOR derivatives tested in this study were modified at position R7 of the quinolone scaffold. Although FQs used today in therapy are generally well tolerated, structure–activity relationship studies have shown that R7 moieties (e.g., piperazine, methylpiperazine, pyrrolidinyl groups) may be linked to central nervous system (CNS) adverse effects of different severity such as dizziness, insomnia, depression, headache, and, rarely, seizures [68,69]. However, it also appears that bulky R7 substituents can decrease such CNS effects [69].

CNS adverse effects of FQs have been shown to be facilitated by the co-administration of NSAIDs and other agents such as theophylline, and have been linked to the low-affinity inhibition of GABA-A receptors and/or to a competitive stimulation of the glutamate/NMDA receptor (for a comprehensive review, see [70,71]). Thus, possible interactions of novel FQs with drugs that may predispose to CNS adverse effects (e.g., NSAIDs, theophylline) should be investigated as well, in the frame of preclinical studies.

## 5. Conclusions

In conclusion, in this work, the superior anticancer activities of novel CIP and NOR derivatives were evidenced in a panel of 2D- and 3D-cultured cancer cell lines, representative of tumors for which therapeutic options are limited, highlighting the possibility of repurposing the modified forms of CIP and NOR as a potential treatment in these types of tumors. Furthermore, we identified some of the mechanisms—among which there was quite a novel one (namely, histone deacetylase inhibition)—that may be at the basis of the improved antineoplastic activity of novel fluoroquinolone derivatives.

Interestingly, despite minor to moderate toxic effects being observed in the fibroblast cell line WH1 [29], all derivatives showed higher potency on the cancer cell lines tested, compared to the fibroblast one (Table S1), indicating that a preferential antiproliferative effect is exerted on cancer cells. We speculate that encapsulation of these molecules within suitably designed delivery systems could overcome such safety-related issues.

## Figures and Tables

**Table 1:** IC_50_ values (μM) obtained following 72h treatment of MCF7, MDA-MD231, MDA-MB453, RT112, and 5637 cells with the new fluoroquinolones and their reference compounds and MTT assay (mean ± S.D. of 4/5 independent experiments; * *p* < 0.05, ** *p* < 0.01, *** *p* < 0.001 vs. CIP; °°° *p* < 0.001 vs. NOR).

	MCF7	MDA-MB231	MDA-MB453	RT112	5637
CIP	10.51 ± 0.97	13.15 ± 1.81	11.43 ± 2.32	9.05 ± 0.73	6.14 ± 0.14
**7** **a**	2.79 ± 0.33 **	1.81 ± 0.21 ***	5.9 ± 1.00 **	1.45 ± 0.26 *	2.44 ± 0.12 *
**7** **b**	3.56 ± 0.75 **	1.88 ± 0.26 ***	3.82 ± 0.50 **	2.02 ± 0.35 *	2.11 ± 0.52 *
NOR	18.11 ± 2.04	20.64 ± 1.81	18.35 ± 2.04	17.41 ± 3.34	37.64 ± 3.3
**7** **c**	3.21 ± 0.77 °°°	1.84 ± 0.30 °°°	2.87 ± 0.68 °°°	2.65 ± 0.84 °°°	2.09 ± 0.74 °°°
**7** **d**	4.17 ± 0.66 °°°	4.08 ± 0.54 °°°	5.65 ± 0.79 °°°	3.62 ± 0.72 °°°	2.34 ± 0.69 °°°

## Data Availability

All data are available upon request.

## Supplementary Materials

The following supporting information can be downloaded at: https://www.mdpi.com/article/10.3390/cancers16122227/s1, Figure S1: Effects of subtoxic concentrations of CIP, NOR, and their derivatives on the migratory capacity of MDA-MB231 (A), MDA-MB453 (B), RT112 (C), and 5637 (D) cells; Figure S2: Representative MMP2 and MMP9 Western blot analysis in MDA-MB231, MDA-MB453, RT112, and 5637 cells following treatment with **7a**, **7b**, **7c**, and **7d** and their precursors CIP or NOR; Figure S3: Densitometric analysis of all Western blot experiments evaluating H3 and H4 protein levels performed in MCF7, MDA-MB231, MDA-MB453, RT112, and 5637 cell lines following 72 h of treatment with **7a**, **7b**, **7c**, **7d**, and their progenitors at concentrations corresponding to the respective IC_50_ values reported in Table 1 (mean ± S.D. of two independent experiments; * *p* < 0.05, ** *p* < 0.01, *** *p* < 0.001 vs. CTR; ° *p* < 0.05, °° *p* < 0.01, °°° *p* < 0.001); Table S1: Potency index of **7a**, **7b**, **7c**, and **7d** compared to their respective progenitors; Table S2: Summary of the increase in acetylated H3 and/or H4 protein levels in MCF7, MDA-MB231, MDA-MB453, RT112, and 5637 cells following 72 h of treatment with CIP, NOR, and their derivatives; Table S3: In silico LogP values for Cip, Nor, and compounds **7a**–**d**.
