# Supplementary material for: Mechanisms of the Antineoplastic Effects of New Fluoroquinolones in 2D and 3D Human Breast and Bladder Cancer Cell Lines
Source: Cancers (Basel). 2024 Jun 14;16(12):2227. doi: 10.3390/cancers16122227 (PMC11201967; doi:10.3390/cancers16122227)
Supplement: Supplementary file 1 [file cancers-16-02227-s001.zip › supplementary figures june 6th.pptx]

## Slide 1
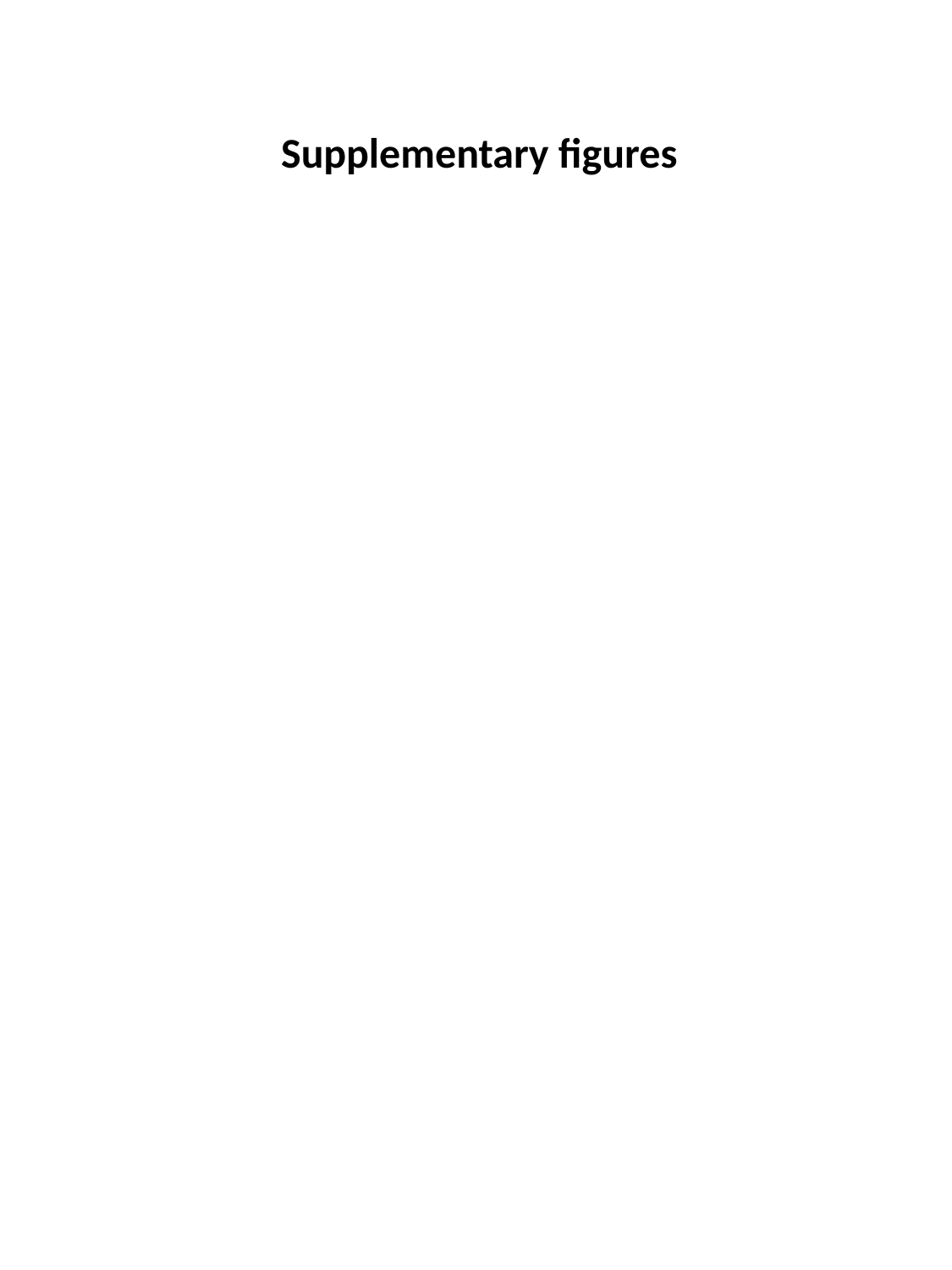

Supplementary figures

## Slide 2
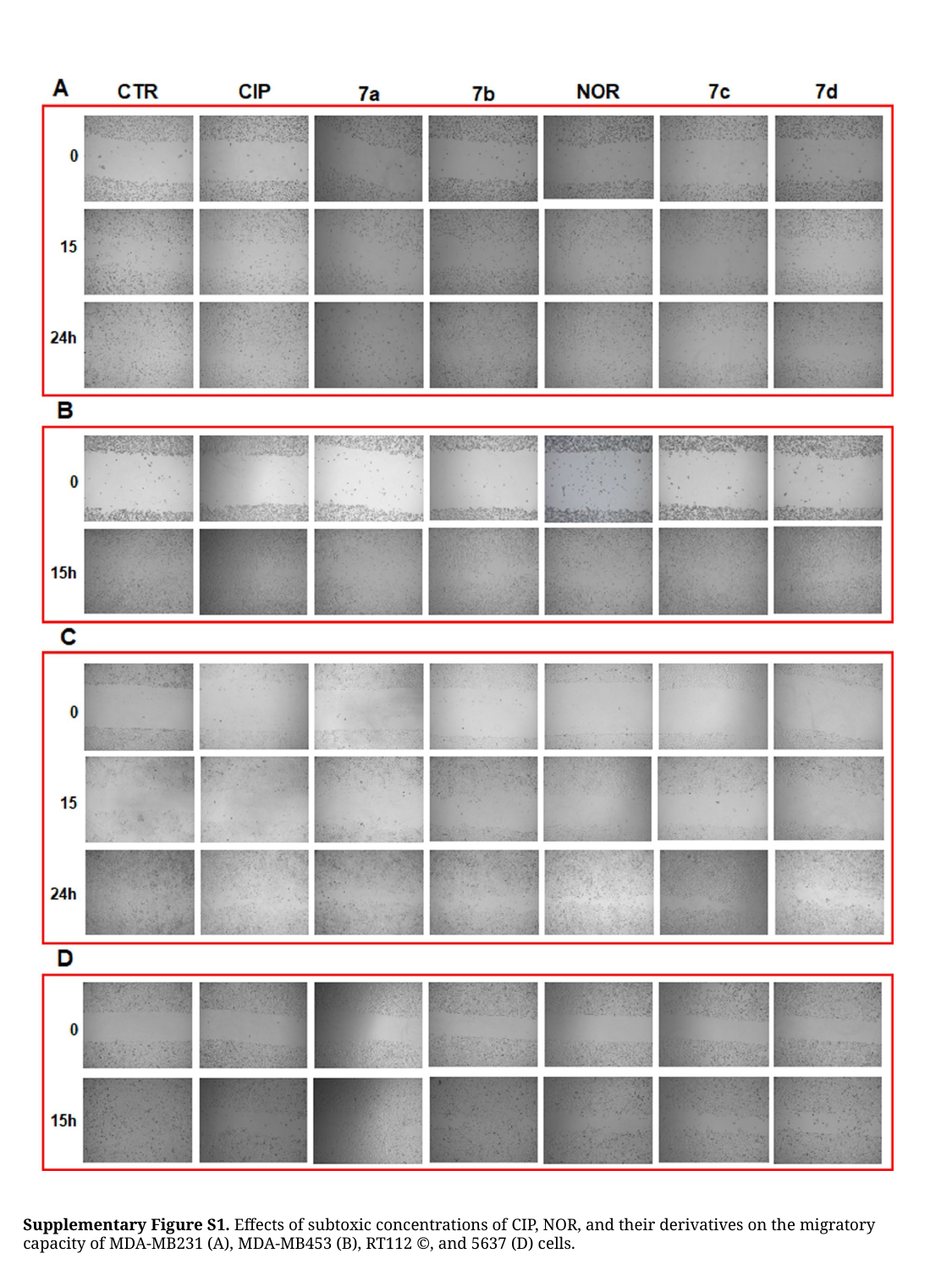

Supplementary Figure S1. Effects of subtoxic concentrations of CIP, NOR, and their derivatives on the migratory capacity of MDA-MB231 (A), MDA-MB453 (B), RT112 ©, and 5637 (D) cells.

## Slide 3
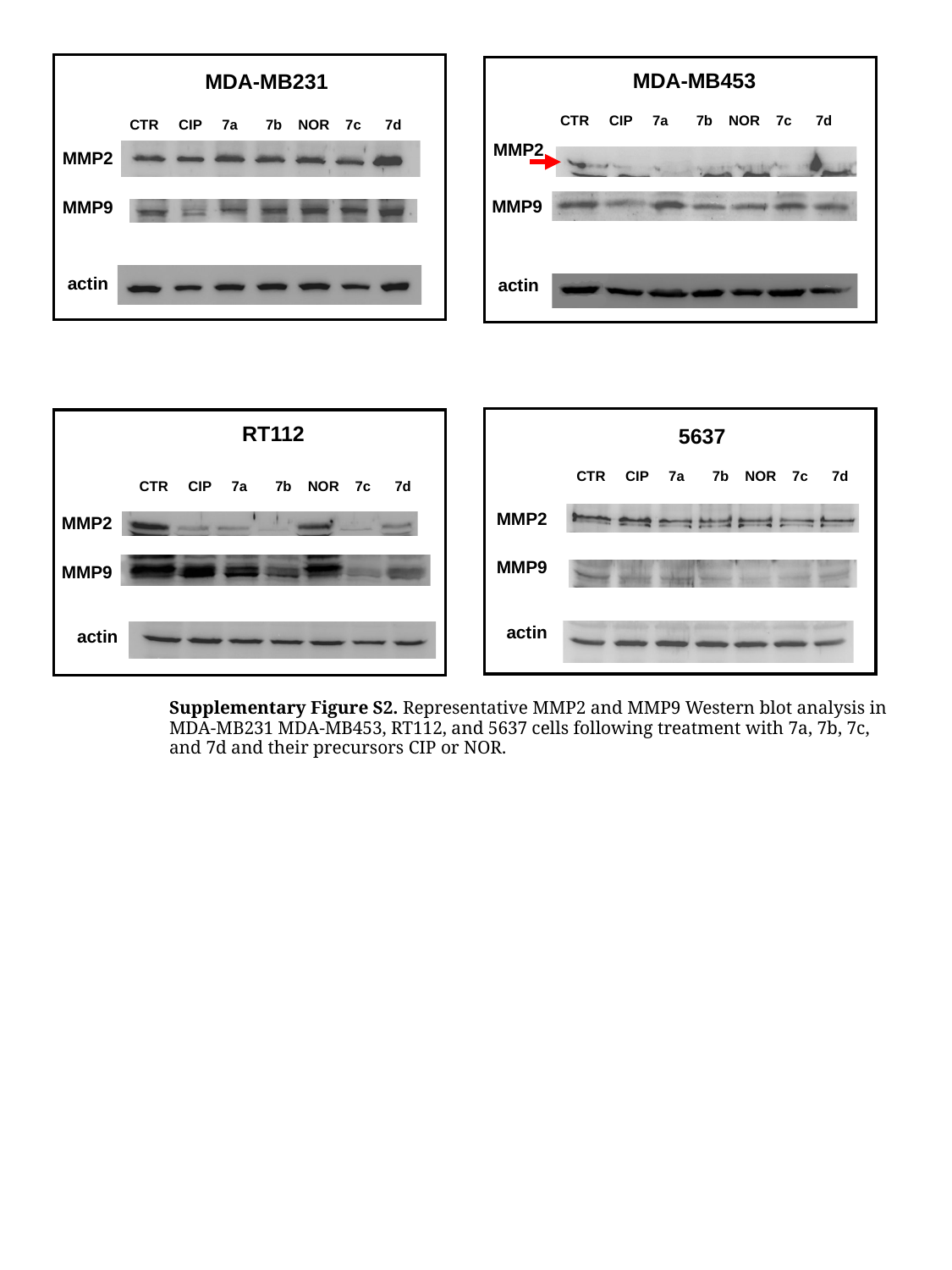

MDA-MB231
CTR CIP 7a 7b NOR 7c 7d
MMP2
MMP2
MMP9
MMP9
actin
MMP2
MMP2
MMP9
MMP9
MDA-MB453
CTR CIP 7a 7b NOR 7c 7d
actin
5637
CTR CIP 7a 7b NOR 7c 7d
actin
RT112
CTR CIP 7a 7b NOR 7c 7d
actin
Supplementary Figure S2. Representative MMP2 and MMP9 Western blot analysis in MDA-MB231 MDA-MB453, RT112, and 5637 cells following treatment with 7a, 7b, 7c, and 7d and their precursors CIP or NOR.

## Slide 4
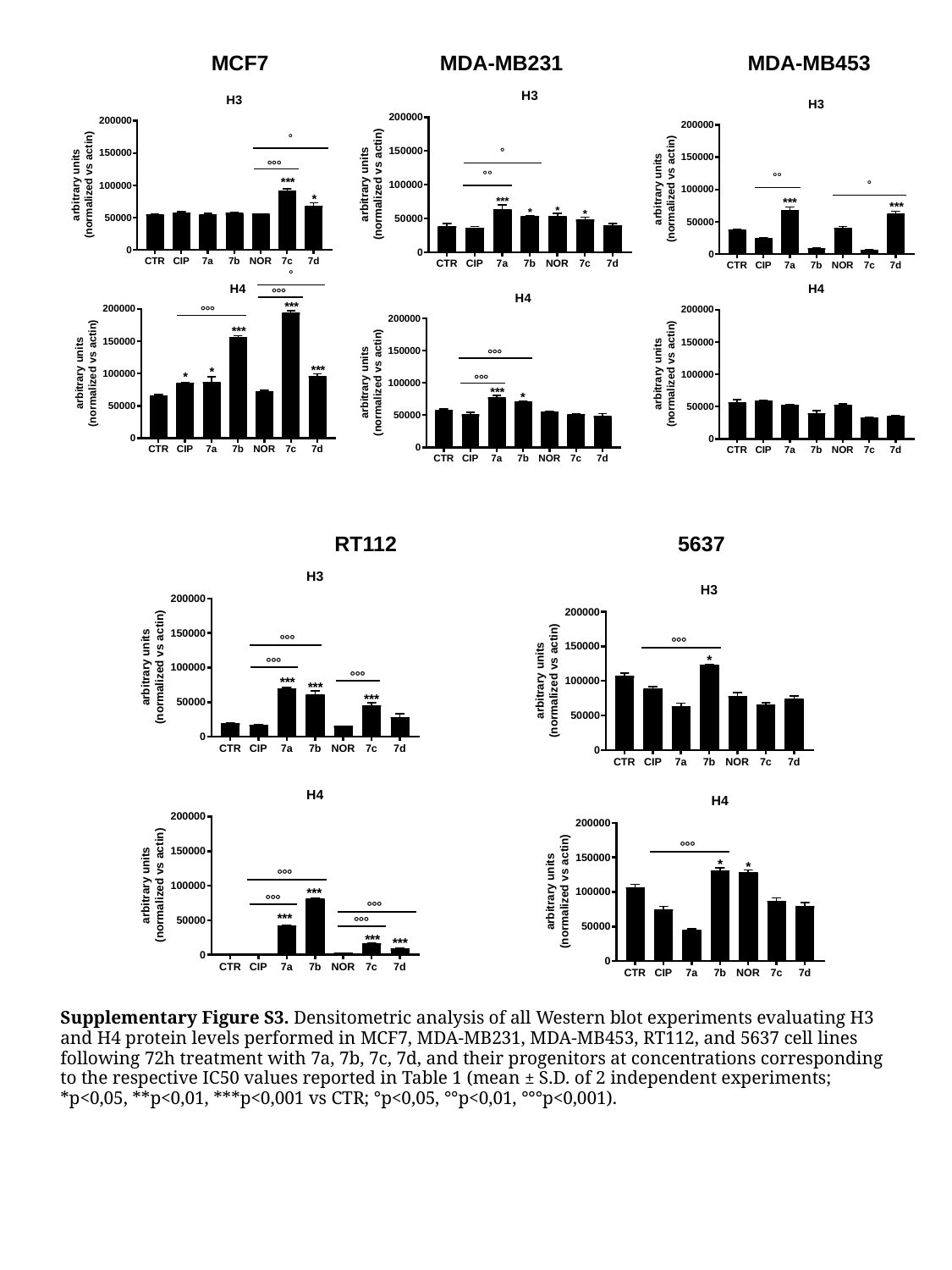

MDA-MB453
MDA-MB231
MCF7
RT112
5637
Supplementary Figure S3. Densitometric analysis of all Western blot experiments evaluating H3 and H4 protein levels performed in MCF7, MDA-MB231, MDA-MB453, RT112, and 5637 cell lines following 72h treatment with 7a, 7b, 7c, 7d, and their progenitors at concentrations corresponding to the respective IC50 values reported in Table 1 (mean ± S.D. of 2 independent experiments; *p<0,05, **p<0,01, ***p<0,001 vs CTR; °p<0,05, °°p<0,01, °°°p<0,001).

## Slide 5
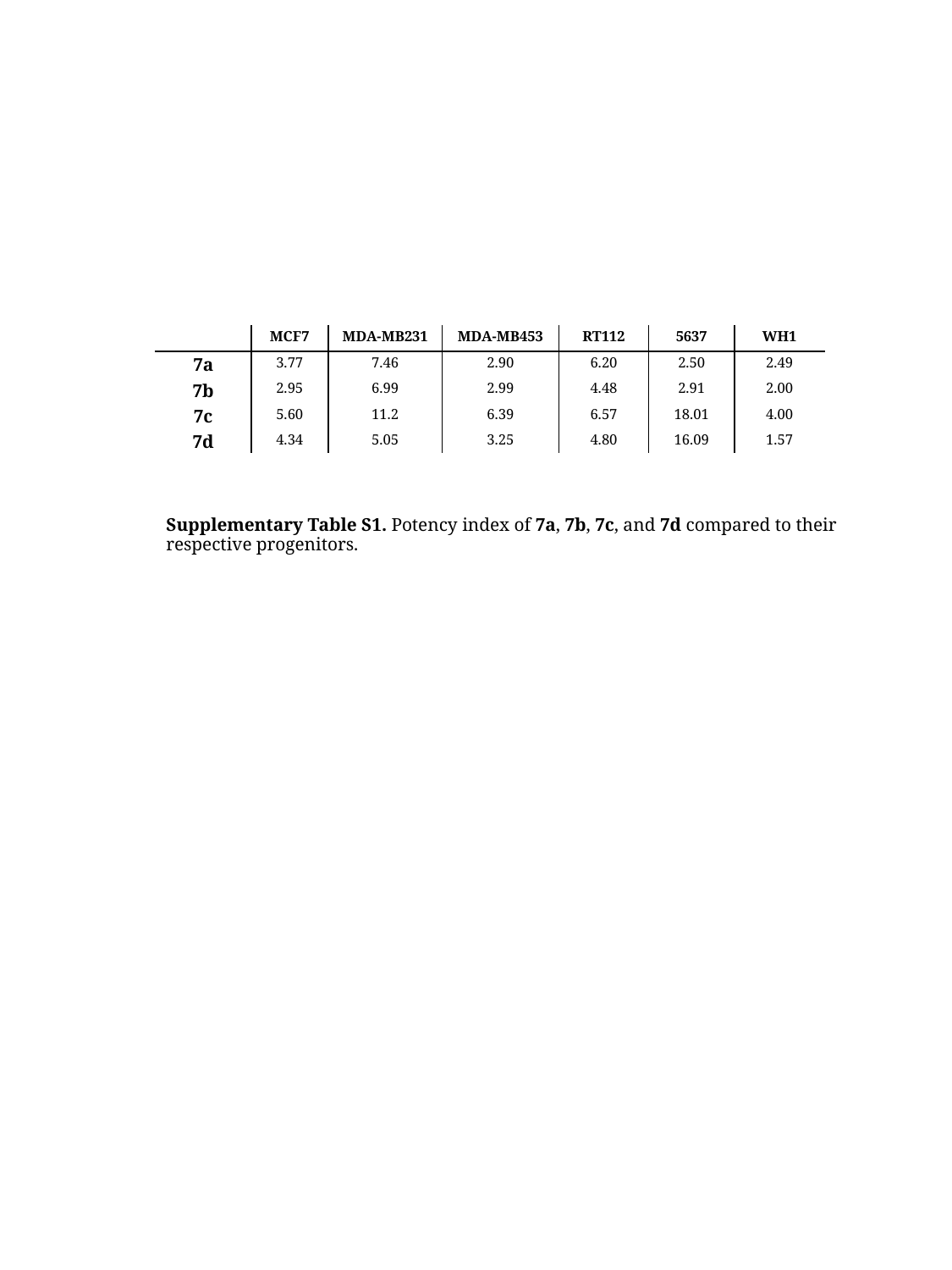

| | MCF7 | MDA-MB231 | MDA-MB453 | RT112 | 5637 | WH1 |
| --- | --- | --- | --- | --- | --- | --- |
| 7a | 3.77 | 7.46 | 2.90 | 6.20 | 2.50 | 2.49 |
| 7b | 2.95 | 6.99 | 2.99 | 4.48 | 2.91 | 2.00 |
| 7c | 5.60 | 11.2 | 6.39 | 6.57 | 18.01 | 4.00 |
| 7d | 4.34 | 5.05 | 3.25 | 4.80 | 16.09 | 1.57 |
Supplementary Table S1. Potency index of 7a, 7b, 7c, and 7d compared to their respective progenitors.

## Slide 6
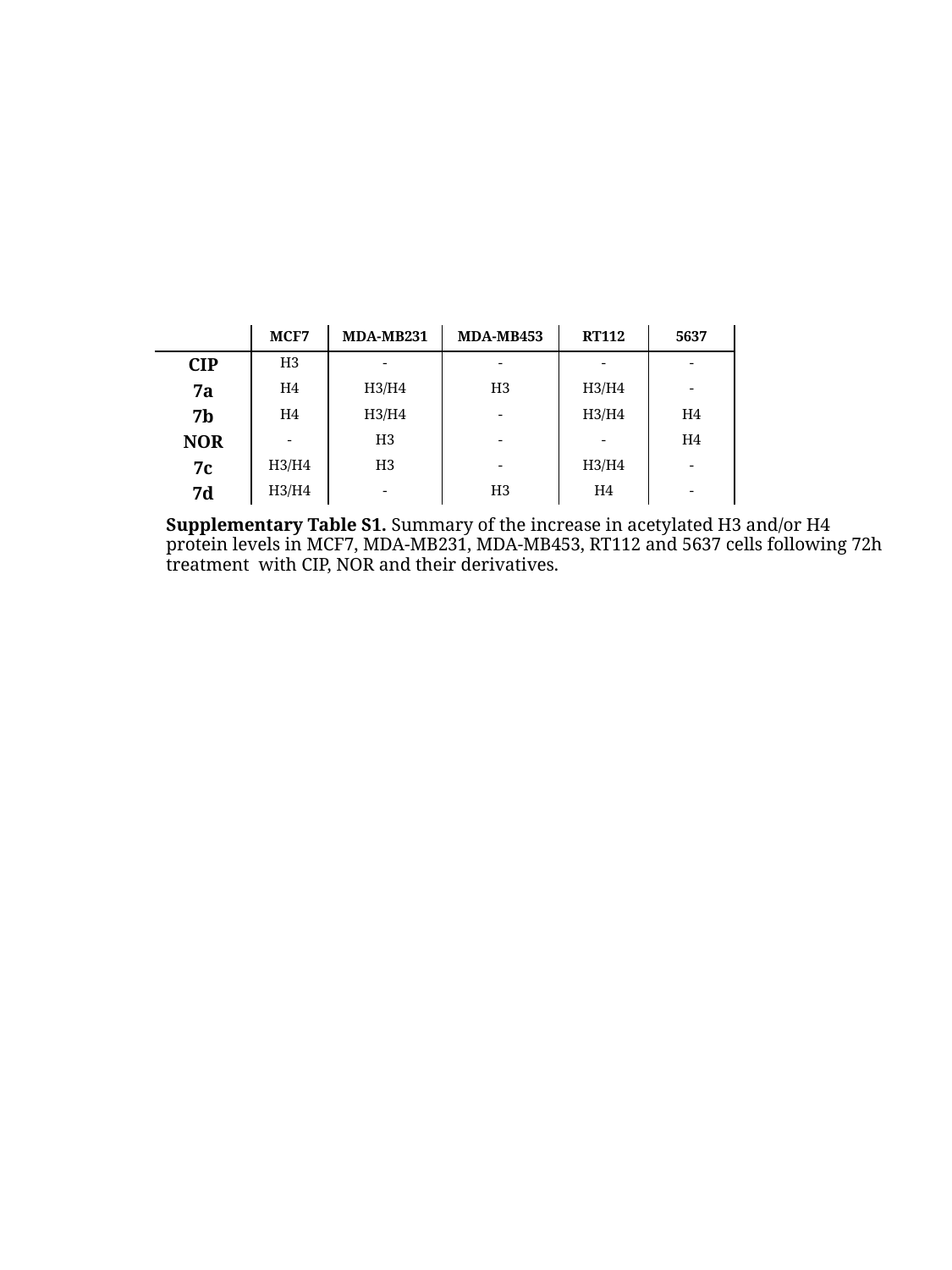

| | MCF7 | MDA-MB231 | MDA-MB453 | RT112 | 5637 |
| --- | --- | --- | --- | --- | --- |
| CIP | H3 | - | - | - | - |
| 7a | H4 | H3/H4 | H3 | H3/H4 | - |
| 7b | H4 | H3/H4 | - | H3/H4 | H4 |
| NOR | - | H3 | - | - | H4 |
| 7c | H3/H4 | H3 | - | H3/H4 | - |
| 7d | H3/H4 | - | H3 | H4 | - |
Supplementary Table S1. Summary of the increase in acetylated H3 and/or H4 protein levels in MCF7, MDA-MB231, MDA-MB453, RT112 and 5637 cells following 72h treatment with CIP, NOR and their derivatives.

## Slide 7
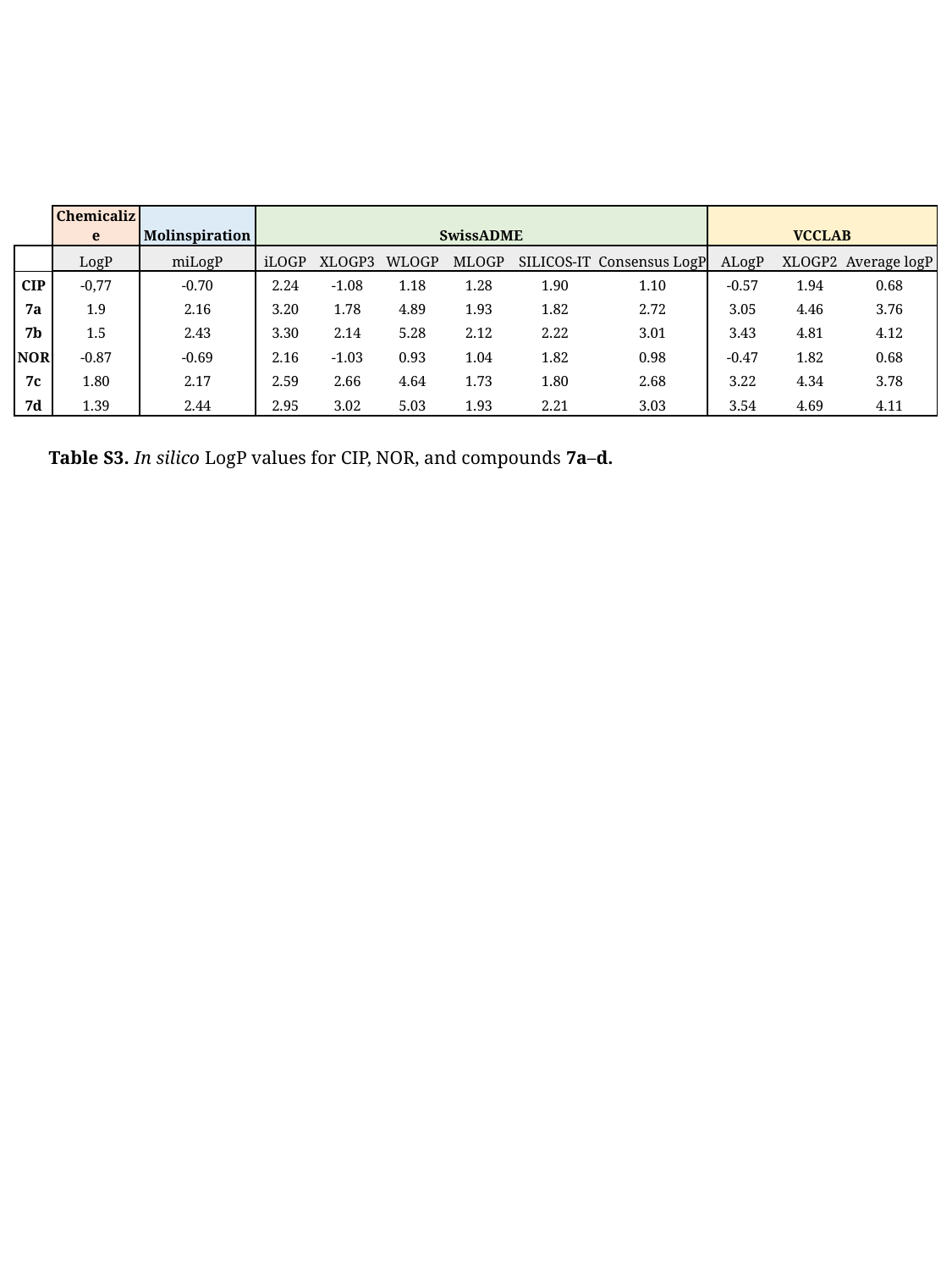

| | Chemicalize | Molinspiration | SwissADME | | | | | | VCCLAB | | |
| --- | --- | --- | --- | --- | --- | --- | --- | --- | --- | --- | --- |
| | LogP | miLogP | iLOGP | XLOGP3 | WLOGP | MLOGP | SILICOS-IT | Consensus LogP | ALogP | XLOGP2 | Average logP |
| CIP | -0,77 | -0.70 | 2.24 | -1.08 | 1.18 | 1.28 | 1.90 | 1.10 | -0.57 | 1.94 | 0.68 |
| 7a | 1.9 | 2.16 | 3.20 | 1.78 | 4.89 | 1.93 | 1.82 | 2.72 | 3.05 | 4.46 | 3.76 |
| 7b | 1.5 | 2.43 | 3.30 | 2.14 | 5.28 | 2.12 | 2.22 | 3.01 | 3.43 | 4.81 | 4.12 |
| NOR | -0.87 | -0.69 | 2.16 | -1.03 | 0.93 | 1.04 | 1.82 | 0.98 | -0.47 | 1.82 | 0.68 |
| 7c | 1.80 | 2.17 | 2.59 | 2.66 | 4.64 | 1.73 | 1.80 | 2.68 | 3.22 | 4.34 | 3.78 |
| 7d | 1.39 | 2.44 | 2.95 | 3.02 | 5.03 | 1.93 | 2.21 | 3.03 | 3.54 | 4.69 | 4.11 |
Table S3. In silico LogP values for CIP, NOR, and compounds 7a–d.
